# Analgesic Medicinal Plants in Shahrekord, Southwest of Iran: An Ethnobotanical Study

**DOI:** 10.31661/gmj.v8i0.1593

**Published:** 2019-09-18

**Authors:** Gholam Basati, Saber Abbaszadeh, Arqavan Zebardast, Hassan Teimouri

**Affiliations:** ^1^Biotechnology and Medicinal Plants Research Center, Ilam University of Medical Sciences, Ilam, Iran; ^2^Razi Herbal Medicines Research Center, Lorestan University of Medical Sciences, Khorramabad, Iran; ^3^Student Research Committee, Lorestan University of Medical Sciences, Khorramabad, Iran; ^4^Hepatitis Research Center, Lorestan University of Medical Sciences, Khorramabad, Iran; ^5^Student Research Committee, Babol University of Medical Sciences, Babol, Iran; ^6^Department of Anesthesiology, Lorestan University of Medical Sciences, Khorramabad, Iran

**Keywords:** Herbal Medicine, Sedatives, Ethnobotany, Shahrekord, Iran

## Abstract

**Background:**

Identification of indigenous medicinal plants, including the gathering of information regarding the uses of these plants can help find out their traditional pharmacological activities and their benefits for the community’s healthcare system. In this study, an ethnobotanical investigation was conducted in Shahrekord city, southwest of Iran to indicate the ethnobotanical knowledge about analgesic medicinal plants in the region and the methods of using them.

**Materials and Methods:**

To this end, plant antioxidants and analgesic medicinal plants were identified. For this purpose, a questionnaire was used to obtain indigenous knowledge from traditional therapists in Shahrekord regarding pain relief using medicinal plants. This ethnobotanical study was conducted in 2018 with the participation of 29 traditional therapists of the region under purpose. Finally, the data drawn from the questionnaires were analyzed using the Excel software. The frequency of plants use was also calculated.

**Results:**

Our study showed that in Shahrekord, 23 species of medicinal plants are used to relieve pain. The highest frequency of use was obtained for *Eugenia caryophylata* (44%), followed by *Alhagi maurorum* (31%), *Tribulus terrestris* (27%), and *angustifolia* (24%). The Laminaceae family (7 species) was the most frequently used plant family for pain relief. The most frequently used plant organ to relieve the pain was flower (25%), followed by the stem (22%) and leaves (19%).

**Conclusion:**

Given the high importance of medicinal plants in Shahrekord, the results of this study and additional scientific investigations can help produce more effective and less harmful drugs from medicinal plants.

## Introduction

Pain is one of the main causes of disability, anxiety, and job loss [1]. Pain refers to an unpleasant sensory experience that is caused by actual or potential tissue damage [2, 3]. In fact, pain serves as a protective mechanism for the body to exhibit an appropriate response to remove the causative agent of pain and is also used for diagnosis of diseases [4]. Analgesics are of two types: opioids and non-opioids [5]. Analgesics relieve pain by disturbing the consciousness or changing other sensory receptors through central or peripheral mechanisms [6]. Opioids are strong analgesics but can lead to substantial side effects. To reduce the harmful effects of opioids, a combination of opioid and non-opioid analgesic, such as acetaminophen or aspirin, is used in most cases. Tramadol is also an opioid analgesic that acts by increasing catecholamine, in addition to activating serotonin receptors, in the central nervous system [7, 8]. Corticosterosids are another class of medicines that reduce pain and inflammation by stopping the inflammatory chain through inhibiting the phospholipase enzyme, but they are used in certain conditions such as immunosuppression and delayed tissue repair [9]. Neurological diseases including anxiety, stress, depression, insomnia, and pain due to various diseases have a relatively high prevalence and decrease the quality of life of the affected individuals [10-12]. Over the past decade, interest in various types of complementary medicine and herbal medicine has been increasing around the world [13-19]. Medicinal plants are a rich source of bioactive ingredients that are used to treat various diseases [20-24], including mental disorders such as anxiety, stress, depression, and various types of pains like headache, migraine, and toothache [23-25]. One of the controversial issues regarding medicinal plants is local knowledge. This knowledge is very extensive and includes various domains, including the ethnobotany of medicinal plants. Ethnobotany refers to the knowledge of humans about the botany and ecology of the plants’ environment. Given the ecological diversity of Iran, identification of indigenous plants across the country and gathering of information regarding the shapes and uses of these plants can help find out their pharmacological activities and their benefits for the community’s healthcare system. In this study, an ethnobotanical investigation was conducted in Shahrekord city, southwest of Iran, to study the ethnobotanical knowledge about analgesic medicinal plants and the methods of using them in the region. Since older people, including traditional therapists, mainly possess this knowledge that may be lost with their death, we enrolled 29 traditional therapists in Shahrekord to collect the information in question.

## Materials and Methods

### Studied region

**Figure 1:**
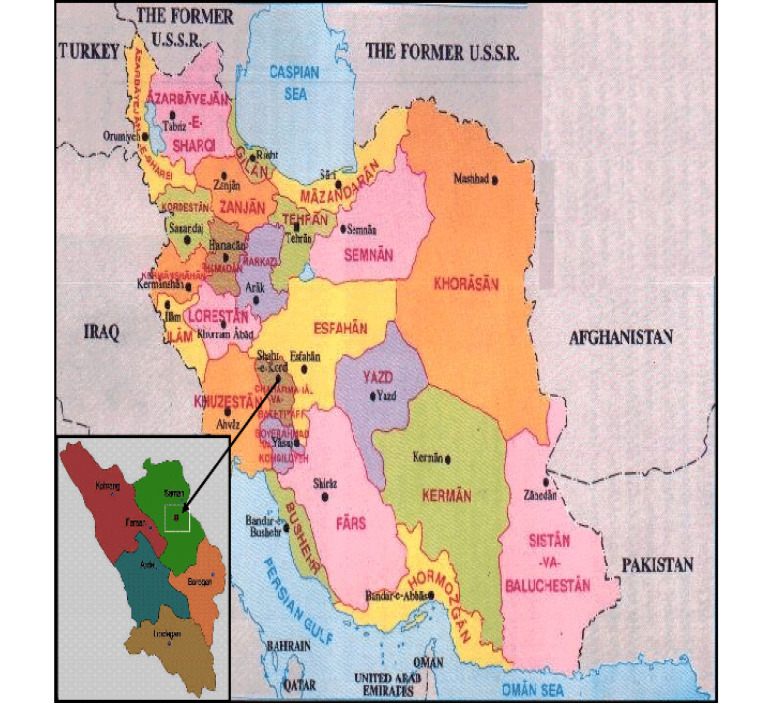
The map of Shahrekord city(southwest of Iran)

Shahrekord city is the capital of Chaharmahal and Bakhtiari province with an area of around 2006 km2, accounting for 12% of the total area of the province. According to the 2011 Iranian Population and Housing Census report, the township of Shahrekord has 283210 inhabitants, of whom 253629 people live in urban areas, and 29581 ones live in rural areas. Three dialects are spoken in the city, i.e., Persian, Lori Bakhtiari, and Turkish. The main occupations of the people of this region are agriculture and animal husbandry. Shahrekord’s map is shown in Figure-1.

### Data Collection

In this ethnobotanical study, traditional knowledge of the participants was collected using a questionnaire in 2018. To this end, the local knowledge of 29 traditional therapists in Shahrekord about the analgesic effects of medicinal plants was drawn with face-to-face interviews and a questionnaire. The questionnaire that had already been prepared was administered to the traditional therapists by the interviewers. The questionnaire included information about the location and demographic characteristics of the respondent and the local name(s) of the plant(s) and the organ(s) used. The interviewers personally referred to the respondents to elicit and record their beliefs about herbal medicine. Among the 29 traditional therapists, eight individuals were female, and 21 ones were male. The education level of the respondents was from high school diploma to master’s degree. The data drawn from the questionnaires were carefully tabulated. Data analysis was performed by the Excel software. In this study, the frequency of plant use was calculated by the equation below:

Frequency of used=(Number of people who have mentioned the plant effect / total number of people who filled out questionnaires) × 100

## Results

**Figure 2:**
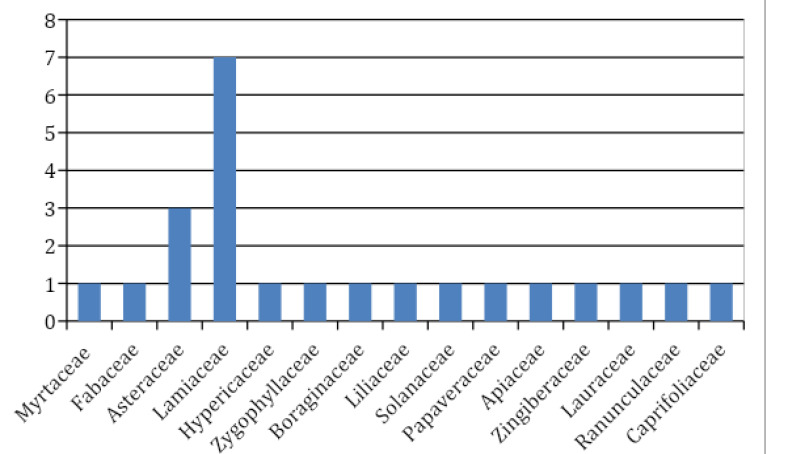
Plant families including species with analgesic effects

The results showed that in Shahrekord, 23 species of medicinal plants are used to relieve pain. As shown in Table-1, the highest frequency of use was obtained for *Eugenia caryophylata* (44%), followed by *Alhagimaurorum* (31%), *Tribulus terrestris* (27%), and *ngustifolia* (24%). Other information is presented in Table-1. The most frequently used organ of the plants for the analgesic property is flower (25%), followed by stem (22%), leaves (19%), aerial organs (14%), seed (11%), root (6%), and bulb (3%). As shown in Figure-2, the Laminaceae family (n=7 species) is the plant family including the highest number of medicinal plants that are used to relieve pain in the studied region.

## Discussion

Plants have always played a substantial role in the health and well-being of human societies. The beneficial effects of the plants, reported in our study, on various diseases have been confirmed by numerous trials [26-32]. *Lavandula officinalis* induces analgesia by affecting inflammatory processes [33]. *Melissa officinalis* has a central analgesic mechanism [34]. *Origanum vulgare L.* shows analgesic effect due to its antioxidant compounds. This plant produces analgesic effect through inflammatory processes and opioid receptors [35]. *Satureja bachtiarica Bung.* exerts its analgesic effect through central mechanisms and inflammatory processes [36]. *Mentha piperita* produces analgesic effect through both central and peripheral effects [37]. *Tanacetum parthenium* exhibits analgesic effect through inflammatory processes [38]. *Hyoscyamus niger* produces analgesic effect through cholinergic and opioid mechanisms [38]. *Cinnamomum verum* exerts analgesic effect through prostaglandins and stimulation of opioid receptors [40]. *Zingiber officinale* produces an analgesic effect by inhibiting the release of peripheral mediators and cytokines [41]. *Anthemis hyalina*DC. also produces analgesic effect through inflammatory processes [42]. Each plant may relieve pain through a specific mechanism of analgesic action. Pain is associated with an increase in oxidative stress, and the antioxidant plant extracts can relieve pain. These and many other plants have been shown to possess antioxidant properties [43-45]. Therefore, they may also act through this mechanism.

## Conclusion

Based on the ethnobotanical knowledge in Shahrekord, several medicinal plants can be used as pain relievers due to certain pharmacological action mechanisms. The sedative and analgesic effects of some of these plants have already been reported. Regarding the difference in the ethnobotany of medicinal plants between the people of Shahrekord and those of other regions across Iran and different uses of different medicinal plants, as well as the availability of certain medicinal plants in the studied area, our findings on the knowledge of the indigenous people in Shahrekord about the use of medicinal plants may be useful. Besides, some medicinal plants used to relieve pain have not yet been adequately studied for this pharmacological activity. Therefore, if this therapeutic approach is chosen, the side effects of the plant(s) must be taken into account because medicinal plants are not completely harmless and their efficacy has not yet been definitely approved. Therefore, they should be investigated in well-designed clinical trials.

## Conflict of Interest

The authors declare no conflict of interest.

**Table 1:** The Scientific Names, Family, Persian Names, Used Organ(S), and Frequency of Use of Medicinal Plants Used as Analgesic in Shahrekord

**Scientific name**	**Herbal family**	**Local name**	**Frequency of use**	**Organ used**
*Eugenia caryophylata*	Myrtaceae	*Mikhak*	44%	Flower, Stem
*Alhagi* *maurorum*	Fabaceae	*Kharshotor*	31%	Root, stem
*Anthemis hyalina DC.*	Asteraceae	*Babouneh*	31%	Flower
*Dracocephalu mmulticaule Montbr & Auch.*	Lamiaceae	*Zarringiah*	3%	Leaf, flower
*Origanum vulgare L.*	Lamiaceae	*Marzanjoush*	13%	Aerial organs
*Thymus vulgaris*	Lamiaceae	*Avishan*	3%	Leaf
*Hypericum scabrum L.*	Hypericaceae	*Goleraei*	3%	Flower, stem
*Tribulus terrestris*	Zygophyllaceae	*Kharkhasak*	27%	Leaf, stem
*Tanacetum polycephalum (L.) Schultz-Bip.*	Asteraceae	*Mokhalaseh*	2%	Leaf, stem
*Anchusa italyca Retz. (L.) DC.*	Boraginaceae	*Gavzaban*	6%	Flower
*Satureja bachtiarica Bung.*	Lamiaceae	*Marzehkouhi*	3%	Aerial organs
*Mentha* *sylvestris*	Lamiaceae	*Naena*	3%	Leaf
*Fritillaria imperialis L.*	Liliaceae	*Lalehvazhgoun*	3%	Bulb
*Lavandula angustifolia*	Lamiaceae	*Ostokhodous*	24%	Aerial organs
*Hyoscyamus kotschyanus Pojark.*	Solanaceae	*Bang daneh*	2%	Seed, leaf
*Papaver rhoeas*	Papaveraceae	*Goleshaghaiegh*	3%	Flower
*Achillea millefolium*	Asteraceae	*Boumadaran*	3%	Aerial organs
*Ferulaovina Boiss.*	Apiaceae	*Koma*	13%	Leaf
*Zingiber officinale*	Zingiberaceae	*Zanjabil*	2%	Root, stem
*Cinnamomum verum*	Lauraceae	*Darchin*	13%	Stem
*Nigella sativa*	Ranunculaceae	*Siahdaneh*	13%	Seed
*Melissa officinalis* L	‏Lamiaceae	*Badranjbouyeh*	3%	Aerial organs
*Valeriana officinalis*	Caprifoliaceae	*Sonbolatie*	3%	Flower, stem
